# Psychological Distress and Quality of Life in Patients with Laryngeal Cancer: A Review

**DOI:** 10.3390/healthcare13131552

**Published:** 2025-06-29

**Authors:** Maria Octavia Murariu, Eugen Radu Boia, Adrian Mihail Sitaru, Cristian Ion Mot, Mihaela Cristina Negru, Alexandru Cristian Brici, Delia Elena Zahoi, Nicolae Constantin Balica

**Affiliations:** 1 Department of Doctoral Studies, “Victor Babes” University of Medicine and Pharmacy Timisoara, Eftimie Murgu Sq. No. 2, 300041 Timisoara, Romania; octavia.brici@umft.ro; 2 ENT Department, “Victor Babes” University of Medicine and Pharmacy Timisoara, Eftimie Murgu Sq. No. 2, 300041 Timisoara, Romania; eugen.boia@umft.ro (E.R.B.); ion.mot@umft.ro (C.I.M.); mihaelaprodea@umft.ro (M.C.N.); balica@umft.ro (N.C.B.); 3 Department of Pediatric Surgery, “Louis Turcanu” Emergency Clinical Hospital for Children, Iosif Nemoianu Street 2, 300011 Timisoara, Romania; adrian.sitaru@umft.ro; 4 Emergency Unit, County Emergency Hospital Resita, 320154 Resita, Romania; alex.brici@gmail.com; 5 Department of Anatomy and Embryology, “Victor Babes” University of Medicine and Pharmacy Timisoara, 300041 Timisoara, Romania

**Keywords:** laryngeal cancer, health-related quality of life, psychological distress, depression, anxiety, total laryngectomy, voice rehabilitation, survivorship care, multidisciplinary approach, barriers to support

## Abstract

Laryngeal cancer significantly affects not only survival but also core functions such as speech, swallowing, and breathing. These impairments often result in substantial psychological distress and reduced health-related quality of life (HRQoL). This review aims to synthesize current evidence regarding the psychological impact, quality of life outcomes, and system-level challenges faced by laryngeal cancer patients while identifying strategies for integrated survivorship care. Anxiety and depressive symptoms are highly prevalent among laryngeal cancer patients, particularly those undergoing total laryngectomy or chemoradiotherapy. HRQoL outcomes vary significantly depending on treatment modality, with long-term deficits noted in domains such as voice, swallowing, and emotional well-being. Access to psychological support and rehabilitation remains inconsistent, hindered by institutional, socioeconomic, and cultural barriers. Structured survivorship models, psychological screening, and patient-centered rehabilitation have demonstrated benefits but are not universally implemented. Comprehensive care for laryngeal cancer must extend beyond tumor control to address persistent functional and psychological sequelae. A multidisciplinary, anticipatory, and personalized approach—centered on integrated rehabilitation and mental health support—is essential to optimize survivorship outcomes and improve long-term quality of life.

## 1. Introduction

Laryngeal cancer is a relatively frequent malignancy of the upper aerodigestive tract and accounts for approximately 2–5% of all cancers worldwide, with an estimated 177,000 new cases and nearly 95,000 deaths annually [1]. While early-stage laryngeal cancer may be managed with organ-preserving approaches such as radiotherapy or partial laryngectomy, advanced-stage disease often necessitates total laryngectomy (TL), resulting in permanent anatomical and functional alterations.

The management of laryngeal cancer varies by tumor stage and includes organ-preserving approaches such as radiotherapy and chemoradiotherapy, as well as surgical options like partial and total laryngectomy. Each modality entails distinct functional consequences—particularly regarding voice, swallowing, and breathing—that are central to patients’ quality of life. These therapeutic differences critically shape the psychological burden experienced and thus provide an important context for interpreting survivorship outcomes [2].

The psychosocial consequences of laryngeal cancer and its treatment are profound. Total laryngectomy leads to the complete loss of natural voice, permanent tracheostomy, and alterations in swallowing, breathing, and social interaction. Even in cases managed with chemoradiotherapy or partial laryngectomy, patients often experience significant impairments in speech intelligibility, communication efficacy, and self-perception [3]. These functional limitations are closely linked to emotional distress, anxiety, depressive symptoms, and social withdrawal [4].

Numerous studies have documented reduced health-related quality of life (HRQoL) in laryngeal cancer patients, with persistent challenges in domains such as speech, social functioning, and emotional well-being [5,6]. Validated instruments such as the European Organization for Research and Treatment of Cancer Head and Neck Questionnaire (EORTC QLQ-H&N35) and the Hospital Anxiety and Depression Scale (HADS) are commonly used to quantify these impacts. Despite these tools and growing research interest, psychological care remains inconsistently integrated into routine oncologic treatment protocols for this population.

The intersection between psychological morbidity and functional disability in laryngeal cancer survivorship is complex and multifactorial, influenced by tumor stage, treatment modality, rehabilitation access, socioeconomic status, and support networks [7]. Recent studies in the literature emphasize that beyond oncological control, survivorship care must encompass proactive mental health screening and targeted psychosocial interventions to improve patient outcomes and reintegration.

This narrative review aims to synthesize the current knowledge on psychological distress and health-related quality of life in patients with laryngeal cancer, highlighting clinical patterns, risk factors, and the need for integrated supportive care approaches.

This is a narrative review based on a qualitative synthesis of the existing literature, selected for its relevance and thematic contribution rather than methodological uniformity. It does not follow a systematic review protocol, allowing for a broader exploration of psychosocial and functional dimensions associated with laryngeal cancer survivorship.

The structure of this review is as follows: Section 2 presents the psychological impact of laryngeal cancer, including prevalence data on anxiety and depression and the influence of treatment modality. Section 3 explores health-related quality of life (HRQoL), detailing assessment tools and outcomes associated with different therapeutic approaches. Section 4 discusses the functional implications of treatment, while Section 5 addresses systemic and individual barriers to psychological care. Section 6 outlines recommendations for integrated survivorship care, and the final section provides general conclusions.

This is a narrative review based on the literature retrieved from PubMed, Scopus, and Web of Science. We searched these databases using combinations of keywords including laryngeal cancer, psychological distress, anxiety, depression, quality of life, survivorship care, and rehabilitation. The inclusion criteria were (1) studies published in English; (2) studies involving adult patients with laryngeal or head and neck cancer; and (3) articles reporting psychological, QoL, or functional outcomes. The exclusion criteria included case reports, letters, and non-peer-reviewed materials. The search covered the literature from January 2004 to March 2025, with a focus on clinically relevant and representative studies.

## 2. Psychological Impact of Laryngeal Cancer

The psychological burden of laryngeal cancer is substantial and multifactorial, arising not only from the existential threat of a cancer diagnosis but also from the profound functional and social disruptions associated with treatment. The anatomical centrality of the larynx in speech, breathing, and swallowing means that therapeutic interventions—particularly total laryngectomy—often result in permanent impairments that challenge patients’ identity, autonomy, and interpersonal relationships. These consequences frequently lead to elevated levels of anxiety, depression, and social withdrawal, significantly reducing the overall quality of life [8,9].

Unlike other malignancies, the disfigurement and loss of vocal communication caused by laryngeal cancer treatments have an immediate and visible impact, making psychosocial adjustment particularly difficult. Patients may struggle with altered self-image, stigmatization due to tracheostomy or impaired speech, and frustration related to loss of independence in social and professional settings. Emotional consequences can be further amplified in those with limited social support, pre-existing psychiatric vulnerabilities, or inadequate access to rehabilitative and psychological care [10].

Recent studies in the literature highlight that psychological distress in laryngeal cancer patients often begins at diagnosis, intensifies during treatment, and persists into survivorship. However, it remains inconsistently recognized and poorly addressed in standard oncology pathways. Validated assessment tools such as the Hospital Anxiety and Depression Scale (HADS) and psychological subscales of the EORTC QLQ-H&N35 are increasingly used in research but are rarely integrated into routine clinical practice. As a result, many patients with clinically significant psychological symptoms go untreated [11,12,13].

### 2.1. Prevalence of Anxiety and Depression

Anxiety and depression are among the most frequently reported psychological comorbidities in patients diagnosed with laryngeal cancer, with substantial implications for both their treatment outcomes and long-term survivorship. The emotional burden is often underestimated in clinical settings despite its high prevalence and documented impact on adherence to treatment, immune function, and quality of life. Multiple studies report that anxiety symptoms affect a significant proportion of patients at diagnosis, with rates varying between 20% and 40% depending on the timing of assessment, diagnostic criteria, and instruments used. A systematic review of head and neck cancer (HNC) patients revealed a baseline anxiety prevalence of 29.9%, which decreased to 17.4% over time, suggesting partial psychological adaptation during survivorship [14,15,16]. However, this trend is not uniform. Anxiety may persist or even intensify during critical phases, such as before major surgery (e.g., total laryngectomy), immediately after discharge, or during speech rehabilitation phases [17].

Depression appears to be equally prevalent and, in some subpopulations, even more pronounced. The prevalence of depressive symptoms among HNC patients varies widely, with estimates ranging from 6% to 42% depending on the population studied and treatment stage [8]. A meta-analysis by Jiménez-Labaig et al. reported higher rates of depressive symptoms in patients undergoing radiotherapy, with peak levels observed during or shortly after the treatment course [14]. These fluctuations indicate the need for dynamic, time-sensitive screening approaches rather than one-time assessments.

More specifically, patients with laryngeal cancer have been found to experience higher rates of depression than those with other head and neck subsites. In a national study involving 71,541 hospitalized patients with HNC, the overall prevalence of major depressive disorder (MDD) was 9.3%, with the laryngeal cancer subgroup demonstrating the highest prevalence at 28.5% [18]. This finding underscores the unique psychological challenges associated with the disease, particularly due to its impact on communication and identity. A comparative overview of selected studies reporting prevalence rates of anxiety and depression in patients with laryngeal or head and neck cancer is provided in Table 1, highlighting the variability in methodology, populations, and psychological assessment tools used.

Sociodemographic factors significantly influence the prevalence of psychological distress in this patient population. The female sex, lower income, marital status (particularly widowed or divorced individuals), and a lack of employment have been associated with higher levels of anxiety and depression [19]. Conversely, patients with strong family support and higher educational attainment tend to demonstrate better emotional resilience. Cultural and healthcare system differences also play a role: studies conducted in countries with limited access to psychological care or voice rehabilitation tend to report higher psychological morbidity [20].

Importantly, psychological distress is not only a comorbidity but can also directly affect oncologic outcomes. Depression has been associated with poorer adherence to treatment, higher symptom burden, and even decreased survival in oncology populations. In laryngeal cancer, where treatment regimens are often prolonged and invasive, untreated depression may impair recovery and lead to premature discontinuation of therapy or reduced participation in rehabilitation [21].

Despite the growing body of literature, routine screening for anxiety and depression remains inconsistently implemented in clinical practice. Instruments like the Hospital Anxiety and Depression Scale (HADS), Patient Health Questionnaire (PHQ-9), and Distress Thermometer are validated tools that can easily be integrated into clinical workflows. Their routine use would allow for early detection and timely referral to mental health professionals. Some studies even advocate pre-treatment psychological assessment as part of the standard diagnostic workup, particularly in patients eligible for total laryngectomy or concurrent chemoradiotherapy [22,23].

In summary, anxiety and depression are highly prevalent and clinically significant among patients with laryngeal cancer. These conditions are influenced by both treatment-related factors and sociodemographic variables, and they can profoundly affect both quality of life and clinical outcomes. A structured approach to psychological screening and intervention is therefore critical, especially in this uniquely vulnerable cancer population.

### 2.2. Impact of Treatment Modalities

The treatment of laryngeal cancer involves multiple modalities, each with distinct implications for physical function and psychological well-being. The most common approaches include radiotherapy, chemoradiotherapy (CRT), partial laryngectomy, and total laryngectomy (TL). While these interventions aim to achieve local control and preserve life, they often result in substantial changes to patients’ communicative abilities, appearance, and daily functioning, which in turn impact their psychological state [24].

Total laryngectomy represents the most radical intervention, involving the complete removal of the larynx, permanent tracheostomy, and irreversible loss of the natural voice. Numerous studies have documented the profound psychological consequences associated with this procedure [2,25]. Johansson et al. found that TL patients experience higher levels of social isolation, frustration, and reduced self-esteem due to difficulties in verbal expression, stigmatization, and altered body image. Loss of vocal identity is particularly distressing as voice plays a central role in personal expression and social interaction. Patients often describe a sense of “losing themselves,” which contributes to heightened levels of anxiety and depressive symptoms [26].

In contrast, organ-preserving treatments such as radiotherapy and chemoradiotherapy may prevent permanent anatomical disfigurement but are not devoid of psychological consequences. Mucositis, dysphagia, xerostomia, and voice alterations are common side effects that persist long after treatment, leading to chronic discomfort, communication challenges, and dietary restrictions. These functional deficits can also interfere with patients’ professional and social roles, contributing to emotional exhaustion and reduced quality of life [27].

Several comparative studies suggest that although patients who undergo TL may report greater initial psychological distress due to the drastic nature of the surgery, they often show better long-term adaptation, particularly when comprehensive rehabilitation programs are provided [28,29]. In contrast, patients treated with CRT may struggle with prolonged and unpredictable side effects, resulting in sustained psychological strain [30]. A longitudinal study by Perry et al. indicated that TL patients who engaged in early voice rehabilitation and had access to psychological support reported levels of psychosocial adjustment comparable to those receiving organ-preserving therapies, suggesting that structured intervention may mitigate initial distress [9].

Furthermore, the mode of communication post-treatment significantly influences psychological outcomes. Patients using tracheoesophageal voice prostheses generally report higher satisfaction and self-confidence than those relying on esophageal speech or electrolarynx devices, which are often associated with a mechanical sound and increased stigma. The availability of speech therapy and familiarity with alternative communication methods play a crucial role in this adjustment [31].

Gender and age may also modulate the psychological impact of treatment. Older patients tend to report greater acceptance of functional loss, whereas younger individuals often experience heightened frustration due to interrupted careers and strained social relationships. Female patients, in particular, may perceive a stronger threat to their body image and self-worth following TL, contributing to higher rates of depression [32].

Importantly, the psychological sequelae of treatment modalities are not only a function of the physical outcomes but also reflect the availability of rehabilitation, emotional support, and pre-treatment psychological preparedness. Studies emphasize the importance of multidisciplinary care, including speech-language therapists, psycho-oncologists, and social workers, to support holistic recovery. However, access to such integrated services remains limited in many settings, further exacerbating disparities in psychological outcomes [33,34].

In conclusion, the psychological impact of treatment modalities in laryngeal cancer is substantial and multifaceted. While total laryngectomy often imposes a more visible and immediate burden, long-term psychological adaptation is possible with adequate support. Conversely, organ-preserving treatments may cause more subtle but persistent challenges that also require dedicated psychosocial care. Individualized treatment planning should therefore consider not only oncological parameters but also the likely psychological trajectory of the patient, emphasizing the need for integrated care pathways that include mental health screening and rehabilitation.

### 2.3. Role of Voice Rehabilitation

Voice rehabilitation is a cornerstone in the post-treatment management of laryngeal cancer, especially in patients who undergo total laryngectomy (TL). Beyond restoring communicative function, voice rehabilitation plays a pivotal role in enhancing psychological well-being, rebuilding self-confidence, and facilitating social reintegration [35].

After TL, the complete loss of natural phonation profoundly affects a patient’s sense of identity and ability to participate in daily social and professional life. The resulting silence can be emotionally devastating, often leading to frustration, social withdrawal, and depressive symptoms [35,36]. However, studies have shown that structured voice rehabilitation significantly mitigates these effects. Peterson et al. conducted a randomized controlled trial which revealed that patients who underwent early and consistent voice rehabilitation reported significantly lower levels of anxiety and depression alongside improved social functioning and general well-being [37].

There are three primary methods of voice restoration: tracheoesophageal speech (TEP), esophageal speech, and the use of an electrolarynx. Among these, TEP is generally considered the gold standard due to its more natural sound quality, ease of learning, and superior outcomes in speech intelligibility [38]. Patients using TEP tend to experience less stigma, better communicative autonomy, and higher satisfaction with their quality of life compared to those using an electrolarynx or esophageal speech. Several studies have corroborated that successful TEP users report significantly fewer symptoms of depression and greater social engagement than non-rehabilitated patients or those using less effective methods [35,39].

The timing of intervention is also crucial. The early initiation of voice rehabilitation—ideally before hospital discharge—has been associated with better long-term psychological outcomes. Preoperative counseling and the demonstration of available speech options have been shown to reduce anticipatory anxiety and improve acceptance of the post-laryngectomy condition [33].

In addition to the technical aspects of voice restoration, the therapeutic alliance formed between the patient and speech-language pathologist (SLP) is a key determinant of psychological adjustment. Consistent, empathetic support from SLPs can help patients overcome initial frustration, build confidence in their new voice, and restore a sense of communicative agency. Moreover, group therapy settings where patients can share experiences and practice communication in a safe environment have been reported to foster peer support and reduce feelings of isolation [40].

However, access to high-quality voice rehabilitation services remains variable across healthcare systems. Geographic disparities, a lack of trained personnel, and limited insurance coverage often pose significant barriers, particularly in low-resource settings. This underscores the importance of integrating speech rehabilitation into national cancer care policies and ensuring equitable access for all patients. It is also essential to consider patient-specific factors such as age, cognitive status, manual dexterity (relevant for managing TEP devices), and personal preferences when selecting the rehabilitation method. Tailoring the approach to individual needs not only increases the likelihood of successful communication but also enhances psychological resilience and autonomy [41].

In summary, voice rehabilitation is not merely a functional intervention—it is a psychosocial lifeline for patients with laryngeal cancer. When implemented effectively, it reduces emotional distress, promotes social reintegration, and restores a sense of agency and identity. Its role should therefore be central in any comprehensive survivorship care plan for laryngectomy patients.

### 2.4. Psychological Interventions

Psychological interventions are increasingly recognized as essential components of comprehensive cancer care, particularly for patients with laryngeal cancer who face unique emotional and communicative challenges. These interventions aim not only to alleviate anxiety and depression but also to support patients in adjusting to life-altering functional losses, such as changes in voice, breathing, and social interaction [42].

The spectrum of psychological interventions includes cognitive-behavioral therapy (CBT), supportive–expressive therapy, mindfulness-based stress reduction (MBSR), psychoeducation, and structured group interventions. Among these, CBT has shown the most consistent evidence in oncology settings. It targets maladaptive thoughts, emotional dysregulation, and behavioral avoidance, which are common in patients confronting disfigurement, communication impairment, and existential distress. Studies have demonstrated that CBT can significantly reduce symptoms of depression and anxiety in head and neck cancer patients, including those with laryngeal cancer, with sustained effects over time [43].

Supportive–expressive therapy provides a safe space for patients to explore their fears, grief, and identity struggles related to their illness and treatment. In laryngeal cancer patients, this form of therapy has been especially beneficial in addressing the psychosocial consequences of total laryngectomy, such as altered body image and loss of vocal self. Facilitating emotional expression through non-verbal means—such as writing, drawing, or technological aids—can help compensate for verbal communication barriers and reduce psychological burden [44].

Mindfulness-based interventions have also gained popularity due to their accessibility and holistic benefits. Programs focusing on present-moment awareness and emotional acceptance have been shown to reduce stress, improve coping, and enhance quality of life in oncology patients. A pilot study in patients with head and neck cancer reported significant reductions in psychological distress following an 8-week mindfulness training program, with improvements being particularly notable in patients with limited social support [44].

Evidence also supports the use of psychoeducational interventions, which aim to provide patients with structured information about their condition, treatment options, coping strategies, and available resources. Empowering patients through knowledge not only reduces anxiety but also increases adherence to treatment and engagement in rehabilitation. For laryngeal cancer patients, psychoeducation tailored to communication alternatives, stoma care, and expectations regarding rehabilitation has proven especially effective in improving psychological outcomes [45].

Group-based therapies offer additional advantages by fostering peer support, normalizing emotional responses, and reducing feelings of isolation. Patients who share experiences with others facing similar challenges often report increased self-efficacy and emotional resilience. However, participation in group sessions may be limited in patients with severe speech impairment, which highlights the need for adaptable formats that accommodate alternative communication methods [46].

A growing body of literature advocates for the integration of psychological services into standard oncologic care. Patients with laryngeal cancer who received psychological counseling during treatment reported significantly lower depression scores and improved health-related quality of life compared to those who did not receive any form of psychological support. These findings underscore the clinical relevance of timely and structured mental health interventions, especially in patients undergoing disfiguring or function-impairing treatments [47].

Despite this evidence, psychological care remains inconsistently implemented in routine oncology practice. Barriers include a lack of trained psycho-oncology personnel, limited institutional resources, and stigma surrounding mental healthcare. Furthermore, clinicians often prioritize tumor control and physical rehabilitation, unintentionally neglecting the psychological dimension of recovery.

Specific psychological interventions with proven benefit in oncology populations include cognitive-behavioral therapy (CBT), supportive–expressive therapy, psychoeducational programs, and mindfulness-based stress reduction (MBSR). CBT has been effective in reducing maladaptive thoughts and improving coping strategies, especially in patients undergoing disfiguring treatments such as total laryngectomy. Group-based therapies also foster peer support and emotional resilience, though participation may be limited in patients with speech difficulties [33,48]. Psychoeducational interventions addressing communication strategies and expectations regarding rehabilitation have shown positive outcomes in laryngeal cancer survivors.

In conclusion, psychological interventions have a demonstrable positive impact on the mental health and overall recovery of laryngeal cancer patients. Their inclusion in multidisciplinary treatment plans is not only beneficial but necessary to address the holistic needs of this vulnerable population. Future efforts should aim to reduce barriers to access and ensure that psychological care is offered proactively, not reactively, throughout the cancer trajectory.

### 2.5. Factors Influencing Psychological Outcomes

The psychological trajectory of patients with laryngeal cancer is influenced by a complex interplay of medical, sociodemographic, and psychosocial factors. Understanding these determinants is essential for identifying at-risk individuals and implementing timely, tailored interventions.

Tumor stage and treatment intensity remain among the most influential medical factors. Advanced-stage tumors frequently require more aggressive interventions, such as total laryngectomy or concurrent chemoradiotherapy, which are associated with greater functional impairment and long-term morbidity. Patients with late-stage disease often experience higher levels of emotional distress due to more extensive body image disruption, prolonged recovery, and a more guarded prognosis. Studies have shown a positive correlation between disease severity and depression scores in laryngeal cancer patients, suggesting that physical disfigurement and treatment sequelae directly contribute to psychological burden [49].

Time since diagnosis is another significant factor. Psychological distress tends to peak around the time of diagnosis and during active treatment but may persist well into survivorship in the absence of appropriate support. Longitudinal studies reveal that while some patients exhibit gradual emotional adaptation, others—particularly those with persistent functional deficits or a lack of social support—develop chronic anxiety or depressive disorders. Late-onset psychological distress may also arise during critical milestones, such as recurrence evaluations or long-term disability reassessment [50,51].

Sociodemographic variables, including age, sex, education level, and socioeconomic status, exert a marked influence on psychological outcomes. Younger patients often report greater emotional disturbance due to the interruption of their careers, family life, and social roles. In contrast, older patients may demonstrate higher levels of psychological resilience but also face challenges related to comorbidities and isolation. Female patients consistently report higher levels of anxiety and depression in oncologic populations, potentially due to increased body image concerns and societal expectations regarding communication and appearance [52].

Socioeconomic status (SES) affects psychological well-being both directly and indirectly. Patients with lower SES frequently have reduced access to rehabilitation services, mental healthcare, and social support networks. Financial toxicity related to cancer treatment can compound psychological distress, particularly in healthcare systems where coverage for speech therapy, prosthetics, and psychological counseling is limited. In some studies, low SES has been associated with increased rates of treatment non-adherence, perceived helplessness, and lower health-related quality of life scores [53].

Social support is one of the strongest protective factors against psychological morbidity. Patients who receive consistent emotional and practical support from family, friends, or caregivers tend to report lower distress and better adaptation to functional changes. Conversely, those living alone, lacking a caregiver, or experiencing strained relationships face a significantly higher risk of depression and social withdrawal. A study by Van Hof et al. showed that caregiver self-efficacy and coping style were directly linked to the patient’s psychological trajectory, underscoring the interconnectedness of patient and caregiver well-being [12].

Pre-existing mental health conditions, such as anxiety disorders, depression, and substance use, also shape the response to cancer diagnosis and treatment. These patients may require intensified psychological support and pharmacologic intervention during their oncologic course. Unfortunately, such vulnerabilities are often underrecognized in standard oncology assessments [54].

Cultural and linguistic factors may affect how patients perceive and express psychological distress, influencing diagnosis and treatment engagement. In some populations, stigma surrounding mental health issues may deter individuals from seeking psychological care, while in others, somatic symptoms may predominate over emotional complaints. This cultural variability underscores the need for culturally sensitive assessment tools and interventions [55].

In summary, the psychological outcomes of laryngeal cancer patients are determined by a multifactorial constellation of influences. Effective mental health support requires not only awareness of these factors but also proactive, individualized screening and intervention strategies. Addressing these determinants in clinical pathways can help in identifying vulnerable patients early on and delivering holistic oncologic care.

In conclusion, the psychological burden experienced by patients with laryngeal cancer is profound, multifactorial, and often underappreciated in routine oncologic care. From the initial diagnosis through treatment and into survivorship, these patients face unique emotional challenges stemming from functional losses, altered identity, and social reintegration difficulties. The type of treatment, the availability of voice rehabilitation, individual coping resources, and systemic factors such as socioeconomic status or support networks all play critical roles in shaping psychological outcomes. Despite growing evidence supporting the value of psychological and rehabilitative interventions, the integration of mental health services into standard care pathways remains inconsistent.

As psychological impact is closely intertwined with patients’ subjective perceptions of well-being, autonomy, and social participation, it directly influences health-related quality of life. The following section will explore in greater depth how laryngeal cancer affects health-related quality of life (HRQoL), highlighting the domains most affected, the tools used to assess them, and the evolving strategies aimed at improving long-term outcomes.

## 3. Health-Related Quality of Life (HRQoL) in Laryngeal Cancer Patients

The assessment of health-related quality of life (HRQoL) has become an essential dimension in the comprehensive care of patients with laryngeal cancer. While oncologic outcomes such as disease-free survival and locoregional control remain critical, they do not fully capture the lived experience of patients coping with the consequences of treatment. In laryngeal cancer, where interventions frequently affect communication, swallowing, breathing, and social functioning, the impact on HRQoL is particularly pronounced [56].

HRQoL encompasses physical, psychological, and social domains of health that are influenced by a person’s experiences, beliefs, expectations, and perceptions. In the context of laryngeal cancer, these domains are often disrupted to a greater extent than in other cancer types due to the centrality of the larynx in daily functioning and identity. The loss or alteration of the voice, for example, extends beyond a mere functional limitation—it affects emotional expression, interpersonal relationships, and vocational reintegration.

This section explores the multidimensional construct of HRQoL in patients with laryngeal cancer, highlighting how it is measured, how different treatment modalities affect outcomes, and which patient-related and contextual factors influence long-term well-being. Understanding HRQoL not only provides valuable insights into patient experiences but also informs shared decision-making, rehabilitation strategies, and the design of patient-centered care pathways.

### 3.1. Concept and Relevance of HRQoL in Oncology

In oncology, HRQoL has gained increasing importance as a complementary outcome measure that helps to evaluate not only the effectiveness of treatment but also its tolerability and acceptability from the patient’s perspective. In the case of laryngeal cancer, HRQoL is especially relevant due to the anatomical and functional role of the larynx in essential activities such as phonation, deglutition, and respiration. Treatments aimed at tumor eradication—whether surgical or non-surgical—often lead to profound changes in these functions, thereby altering patients’ self-image, communication ability, social engagement, and mental health. As a result, an HRQoL assessment is not merely an academic exercise but a necessary tool for understanding how patients adapt to life during and after treatment [57].

The inclusion of HRQoL measures in clinical trials and routine care offers several benefits. It facilitates patient-centered decision-making by highlighting the trade-offs between treatment efficacy and quality of life outcomes. It can also serve as a prognostic indicator; lower HRQoL scores have been associated with reduced adherence to treatment, higher symptom burden, and even decreased overall survival in some cancer populations. Furthermore, HRQoL data can guide the development of supportive interventions aimed at mitigating long-term functional and emotional sequelae [56].

For laryngeal cancer patients, HRQoL domains such as communication, social functioning, emotional well-being, and social participation are particularly vulnerable. This is especially true in patients who undergo total laryngectomy, where permanent anatomical alterations often result in lifelong challenges. Therefore, the systematic evaluation of HRQoL in this population provides essential insights for optimizing rehabilitation strategies, psychological support, and survivorship planning. As modern oncology moves toward a more holistic and patient-centered paradigm, HRQoL is increasingly being integrated into treatment algorithms, clinical guidelines, and health policy frameworks. In laryngeal cancer, where the balance between oncologic control and the preservation of quality of life is especially delicate, HRQoL must be regarded as a core outcome alongside traditional clinical metrics [57].

### 3.2. Assessment Tools Used in Laryngeal Cancer

The evaluation of health-related quality of life (HRQoL) in patients with laryngeal cancer requires the use of validated, multidimensional tools that capture the complex interplay between physical, emotional, and social domains. These instruments are crucial for both clinical research and routine practice as they provide standardized data on patient experiences, treatment impact, and rehabilitation progress. In recent decades, several HRQoL questionnaires have been developed and validated specifically for cancer patients, and some have been adapted or extended to capture the unique challenges faced by those with head and neck malignancies [58].

One of the most widely used instruments is the European Organization for Research and Treatment of Cancer Quality of Life Questionnaire Core 30 (EORTC QLQ-C30), which evaluates general aspects of quality of life across cancer types. It includes subscales assessing physical functioning, emotional functioning, fatigue, pain, and global health status. However, because it lacks specificity for anatomical sites, the QLQ-C30 is often supplemented with disease-specific modules [59].

For laryngeal cancer patients, the EORTC QLQ-H&N35 module is the most commonly used supplement. Designed specifically for head and neck cancer, it includes additional domains such as speech problems, swallowing difficulties, sensory deficits, social eating, and social contact. The speech-related items in the H&N35 are particularly relevant for patients undergoing total laryngectomy or radiotherapy affecting vocal function. Studies have confirmed the psychometric reliability and sensitivity of this tool in capturing both short- and long-term quality of life impairments in laryngeal cancer survivors [60].

Another widely adopted tool is the Hospital Anxiety and Depression Scale (HADS), which focuses on psychological distress. While not a comprehensive HRQoL questionnaire, it provides valuable insights into the emotional domain and is often used alongside HRQoL instruments to contextualize patient responses. Given the high prevalence of anxiety and depression in laryngeal cancer patients, the HADS remains a practical and time-efficient screening tool in both research and clinical settings [61].

The Voice Handicap Index (VHI) is a specialized tool that assesses the functional, physical, and emotional impacts of voice disorders. Although initially developed for general voice pathology, it has been validated in laryngectomized patients and those receiving organ-preserving treatment. The VHI is particularly valuable for assessing the subjective burden of voice-related limitations, offering a voice-specific dimension that may not be fully captured by broader HRQoL instruments [62].

Additional tools such as the Short Form Health Survey (SF-36) and Functional Assessment of Cancer Therapy-Head and Neck (FACT-H&N) are also used in selected studies. The SF-36, while generic, allows for a comparison with general population norms, whereas the FACT-H&N includes items targeting communication, appearance, and social concerns relevant to head and neck cancer [63].

Despite the availability of these instruments, their implementation in clinical practice remains inconsistent. Barriers include time constraints, a lack of training in interpretation, and limited integration into electronic health records. Moreover, there is no universal consensus on which instrument should be routinely used for laryngeal cancer patients, leading to heterogeneity in outcome reporting across studies.

While these instruments provide valuable insights into patient-reported outcomes, it is important to acknowledge certain methodological limitations in the literature. Many of the cited studies rely on self-reported questionnaires, which are inherently subject to response bias, recall bias, and social desirability bias. For instance, patients may underreport symptoms such as depression or functional limitations due to stigma or a misunderstanding of questionnaire items. Additionally, selection bias is common in studies recruiting from specialized tertiary centers, where access to rehabilitation and psychosocial support may not reflect broader clinical settings [64].

Some studies also exhibit heterogeneity in inclusion criteria, use different instruments to measure the same constructs, or lack longitudinal follow-up, making comparisons across populations or time points difficult. These factors should be considered when interpreting reported prevalence rates or HRQoL trajectories. Future research should aim to standardize outcome measures, include more diverse populations, and adopt mixed-methods designs that integrate qualitative and objective assessments [10,12].

A critical appraisal of the included studies reveals several strengths, including the use of validated psychological instruments (e.g., HADS and EORTC QLQ-H&N35) and longitudinal designs in some cohorts. However, limitations include small sample sizes, heterogeneity in outcome measures, limited generalizability due to single-center designs, and a lack of standardized assessment timepoints. Moreover, many studies fail to stratify findings by treatment type, limiting nuanced interpretation [9,13].

In conclusion, multiple validated instruments are available to assess HRQoL in patients with laryngeal cancer. The combined use of general, site-specific, and symptom-targeted tools offers the most comprehensive understanding of patient well-being. Standardizing the use of these tools in clinical pathways would enable more consistent monitoring, better communication between providers and patients, and improved tailoring of supportive interventions.

### 3.3. HRQoL Outcomes According to Treatment Modality

The choice of treatment modality in laryngeal cancer profoundly influences health-related quality of life (HRQoL) as each approach imposes distinct functional, esthetic, and psychosocial consequences. While the primary therapeutic goal remains tumor control, preserving or restoring quality of life has become an equally critical objective, especially in early-stage disease where multiple curative options may be available.

Total laryngectomy (TL), typically indicated in advanced or recurrent cases, is associated with some of the most significant long-term impairments in HRQoL. The procedure results in permanent loss of natural voice, altered airway anatomy, and a visible tracheostoma, which can compromise communication, self-image, and social integration. Studies consistently show that patients undergoing TL report lower scores in HRQoL domains related to speech, body image, and social contact. A study using the EORTC QLQ-H&N35 found that TL patients scored significantly worse in the speech, senses, and social eating domains compared to those treated with radiotherapy or chemoradiotherapy. However, when voice rehabilitation is successful—especially via tracheoesophageal puncture (TEP)—some of these deficits can be partially mitigated [65]. Although organ-preserving treatments such as radiotherapy (RT) and chemoradiotherapy (CRT) maintain laryngeal anatomy, they are frequently associated with long-term complications like mucositis, xerostomia, dysphagia, and odynophagia, which significantly impact nutrition, communication, and overall quality of life (HRQoL). While patients treated with CRT may benefit from improved body image and reduced stigma compared to those undergoing total laryngectomy (TL), many still report persistent voice and swallowing difficulties due to fibrosis or edema [66]. A summary of the quality of life instruments used and selected outcomes reported in recent studies is provided in Table 2, illustrating methodological diversity and key findings.

Comparative studies indicate that TL patients often experience a sharper decline in HRQoL shortly after surgery but may adapt better over time—particularly when voice rehabilitation and psychosocial support are available. Conversely, CRT patients often face a delayed but progressive deterioration in function, which may become equally disabling. Voice-related quality of life remains a major concern across all treatment groups. TL patients using tracheoesophageal prostheses generally report higher satisfaction than those relying on esophageal speech or electrolarynx devices. Meanwhile, CRT patients may retain their vocal cords but still struggle with reduced vocal clarity and endurance [28].

Importantly, psychological and social well-being do not depend solely on anatomical preservation. Even patients who avoid laryngectomy may experience significant emotional distress if functional impairments persist. These findings highlight the need for tailored pre-treatment counseling that considers both anatomical and functional outcomes, aiming to align expectations and improve long-term adaptation [67].

In summary, each treatment modality for laryngeal cancer carries a distinct profile of HRQoL consequences. While organ-preserving strategies may offer advantages in body image and initial communicative function, they are often accompanied by persistent toxicity that can impair quality of life over time. Conversely, although TL imposes significant physical and social changes, long-term HRQoL may be optimized with adequate rehabilitation. Treatment planning should therefore integrate HRQoL considerations, encouraging shared decision-making and personalized rehabilitation pathways.

### 3.4. Longitudinal Changes in HRQoL

In the acute phase of treatment—whether surgery, radiotherapy, or chemoradiotherapy—HRQoL typically declines due to immediate functional losses and treatment-related toxicity. Patients frequently report increased fatigue, pain, mucositis, dysphagia, and emotional distress during this period. Studies using the EORTC QLQ-C30 and QLQ-H&N35 instruments demonstrate that most functional and symptom scales significantly deteriorate during the first 3 months of treatment, reflecting both physical burden and psychological strain [68].

Following the completion of treatment, a partial recovery phase generally occurs within 6 to 12 months. Physical symptoms such as mucositis and acute inflammation tend to resolve, and some patients begin to adapt to new speech mechanisms or swallowing patterns. Emotional adjustment also improves, especially in individuals who engage in structured rehabilitation or benefit from social support. However, not all domains recover at the same pace. Communication difficulties, social eating, and sensory deficits (e.g., taste or smell alterations) often persist beyond one year post-treatment, particularly in those treated with chemoradiotherapy or total laryngectomy [69].

In long-term survivorship (beyond 12–24 months), HRQoL trajectories vary widely. Some patients achieve near-baseline quality of life, especially if their functional outcomes are favorable and they have adapted psychologically. Others, however, continue to experience significant impairments. Chronic symptoms such as xerostomia, fibrosis, speech limitations, and stoma-related issues can have a cumulative impact on daily functioning [70]. A longitudinal study by Perry et al. showed that even after two years, patients who had undergone total laryngectomy continued to report lower scores in speech, social functioning, and emotional well-being compared to population norms, although their global HRQoL showed signs of stabilization [9].

Patients treated with organ-preserving modalities may report fewer early physical impairments but are not exempt from long-term deficits. For example, persistent dysphonia, fatigue, or swallowing issues may emerge or intensify months after treatment, leading to what some researchers describe as a “delayed decline” in HRQoL. This underscores the need for continued monitoring and late-onset symptom management even in patients who initially appear to have favorable outcomes [71].

Psychological resilience and adaptation play a significant role in shaping long-term HRQoL. Patients who engage in active coping, maintain social relationships, and participate in rehabilitation are more likely to experience a gradual improvement in emotional and social domains. In contrast, those with pre-existing mental health conditions, inadequate support, or unaddressed functional deficits are more prone to prolonged HRQoL deterioration [72].

**Figure 1:**
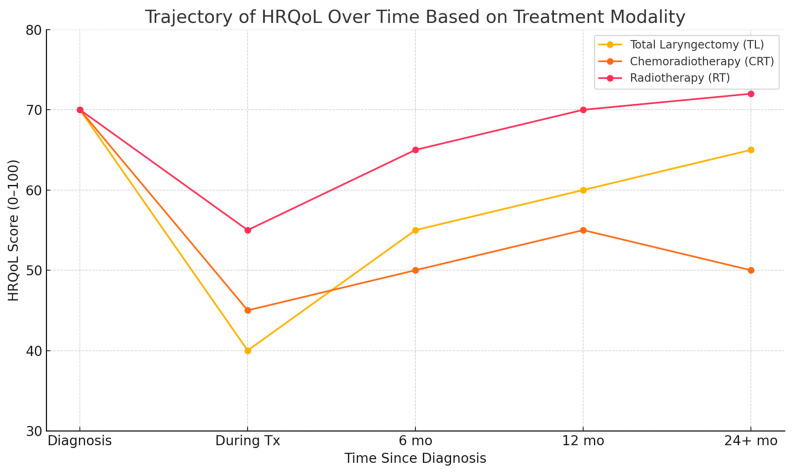
The conceptual trajectory of health-related quality of life (HRQoL) over time in laryngeal cancer patients based on treatment modality. This schematic summarizes trends from selected longitudinal studies and meta-analyses [9,10,11,14] and is intended as an illustrative model, not a representation of pooled or original data. Tx-Treatment; mo-months.

Importantly, HRQoL does not always follow a linear trajectory. Recurrence anxiety, comorbidities, and major life events can trigger fluctuations even years after treatment completion. As such, survivorship care must be dynamic, responsive, and personalized to the evolving needs of each patient. Figure 1 presents a conceptual synthesis of findings reported across multiple longitudinal studies investigating HRQoL trajectories in laryngeal cancer. The illustrated trends are based on comparative data from studies by Johansson et al. (2011) [10], Perry et al. (2015) [9], and Bergström et al. (2017) [11] and the meta-analysis by Jiménez-Labaig et al. (2025) [14]. This figure is a conceptual illustration synthesized from multiple longitudinal studies and meta-analyses. It does not present original or pooled data but rather integrates generalized trends reported in the literature. Its purpose is to support narrative synthesis and should be interpreted accordingly. Limitations include variability in study design, the HRQoL instruments used, and population characteristics across the referenced sources.

In summary, HRQoL in laryngeal cancer patients changes significantly over time, with most individuals experiencing initial decline, partial recovery, and variable long-term adjustment. While some domains improve with time and support, others may remain chronically affected. Longitudinal assessment is therefore vital to guide rehabilitative strategies, inform clinical follow-up, and support patients across the entire cancer continuum.

## 4. The Role of Treatment Modality

Building on the previously discussed psychological and quality of life outcomes, this section focuses on the clinical implications of treatment modality selection and its role in shaping functional recovery, rehabilitation needs, and patient-centered care strategies.

Treatment modality in laryngeal cancer represents a critical determinant not only of oncologic control but also of functional recovery, long-term autonomy, and reintegration into daily life. Therapeutic strategies are selected based on tumor stage, anatomical considerations, and patient comorbidities, yet they vary significantly in their impact on voice preservation, swallowing efficiency, and airway management. As clinical protocols evolve, the emphasis shifts toward balancing survival outcomes with functional conservation and patient-centered goals [73]. Understanding the nuances of each treatment option—beyond efficacy—allows clinicians to tailor interventions that align with individual priorities and support holistic recovery. This section reviews the spectrum of therapeutic approaches used in laryngeal cancer and explores how treatment choice influences functional trajectories and overall patient experience.

### 4.1. Overview of Therapeutic Options

The therapeutic landscape of laryngeal cancer includes a range of interventions that differ in their aggressiveness, functional outcomes, and applicability across tumor stages. Primary modalities include surgery (either organ-preserving or ablative), radiotherapy, and concurrent chemoradiotherapy. Each of these options can be used alone or in combination depending on the location, extent, and biological behavior of the tumor.

Early-stage laryngeal cancers (T1–T2 and N0) are typically managed with single-modality therapy, either radiotherapy or transoral laser microsurgery (TLM). Both approaches offer high rates of local control while aiming to preserve voice and swallowing function. Radiotherapy has the advantage of being non-invasive and avoids anesthesia-related risks, while TLM allows for a histopathological margin assessment and may offer shorter treatment durations. Selection between these two is often based on tumor accessibility, patient comorbidities, and preferences regarding potential voice outcomes [74,75,76].

Locally advanced disease (T3–T4 or node-positive tumors) generally requires more complex regimens. Concurrent chemoradiotherapy (CRT) has become a cornerstone in organ-preserving protocols for advanced laryngeal cancer. This approach combines radiotherapy with platinum-based chemotherapy to enhance radiosensitivity and achieve tumor control without resorting to total laryngectomy. CRT is particularly valuable in patients who express a strong preference for voice preservation. However, it is associated with cumulative toxicity, including mucositis, weight loss, and risk of treatment-related complications that may necessitate enteral feeding or tracheostomy [77].

Surgical options are often reserved for cases where organ preservation is not feasible or has failed. Total laryngectomy (TL) remains the definitive curative procedure for advanced-stage or recurrent disease. While it provides excellent local control and eliminates tumor bulk entirely, TL necessitates permanent anatomical alteration, including removal of the vocal cords and the creation of a tracheostoma. Alternatively, partial laryngectomy techniques, such as supraglottic or vertical hemilaryngectomy, may be employed in select intermediate-stage tumors to achieve oncologic safety while retaining partial phonatory and protecting laryngeal functions. In some instances, salvage surgery becomes necessary after CRT failure. These procedures are often technically complex due to tissue fibrosis and altered anatomy and may result in higher morbidity. Postoperative complications such as fistula formation, poor wound healing, and dysphagia are more common in irradiated fields, further impacting recovery [48,78].

The diversity of therapeutic options underscores the need for multidisciplinary evaluation, where input from surgical oncologists, radiation oncologists, speech-language pathologists, and psycho-oncology specialists can ensure a personalized and evidence-based treatment plan. Importantly, the choice of modality is not merely a clinical decision but a deeply personal one, influenced by the patient’s communication needs, social roles, and expectations regarding recovery and quality of life.

In recent years, emerging treatment modalities such as immunotherapy and transoral robotic surgery (TORS) have entered the therapeutic landscape for selected cases of laryngeal and hypopharyngeal cancer. Although their primary aim remains oncologic control with improved functional preservation, the psychological impact of these therapies warrants further exploration [79].

Immunotherapy, particularly immune checkpoint inhibitors (e.g., anti-PD-1 agents), has shown promise in recurrent/metastatic head and neck squamous cell carcinoma. Patients undergoing immunotherapy often report less acute toxicity compared to chemoradiation, which may translate into lower treatment-related psychological distress. However, the uncertainty of response, the need for prolonged monitoring, and the novelty of adverse event profiles (e.g., immune-related fatigue and endocrine effects) may generate anxiety or confusion, especially in patients with limited health literacy [80].

Transoral robotic surgery (TORS) is another minimally invasive option that may preserve key laryngeal structures while reducing hospitalization and recovery time. Preliminary data suggest more favorable functional outcomes and faster return to oral intake, which could positively influence postoperative psychological well-being. Nonetheless, access to TORS remains limited, and expectations must be carefully managed to avoid disappointment if long-term voice or swallowing outcomes fall short [81].

Future studies should more explicitly address how these evolving treatments affect psychological adaptation, health-related quality of life, and patient-reported outcomes, especially in comparison with traditional modalities. Future research should aim to (1) standardize psychological assessment protocols in laryngeal cancer care; (2) evaluate the long-term impact of emerging therapies (e.g., immunotherapy and TORS) on mental health; and (3) develop and validate telemedicine and digital interventions tailored for post-laryngectomy rehabilitation.

### 4.2. Impact on Functional Outcomes

The functional consequences of treatment in laryngeal cancer are highly modality-dependent and influence the patient’s ability to perform vital tasks such as speaking, swallowing, and breathing. While survival remains the primary endpoint of oncologic therapy, preserving or restoring these core functions is critical for maintaining autonomy and ensuring long-term recovery.

Voice function is among the most visibly and socially impactful domains. In patients undergoing total laryngectomy, the removal of the vocal folds eliminates natural phonation entirely. While prosthetic voice rehabilitation is possible, speech remains non-physiological and often requires significant adaptation. In contrast, patients treated with radiotherapy or concurrent chemoradiotherapy may retain the anatomical structures necessary for voice production. However, radiation-induced fibrosis, edema, and mucosal changes can lead to chronic dysphonia, reduced vocal range, or voice fatigue. Even in cases of anatomical preservation, vocal quality may be significantly altered, particularly in individuals whose baseline voice demands were high (e.g., teachers and singers) [82].

Swallowing function (deglutition) is also variably affected depending on treatment modality. Radiotherapy and chemoradiotherapy can cause acute mucositis and long-term fibrosis of the pharyngeal constrictors, leading to dysphagia and aspiration risk. The need for enteral feeding, either temporarily or permanently, is not uncommon, especially in the context of concurrent chemotherapy. In surgical cases, particularly following partial laryngectomy, patients may initially experience impaired airway protection due to the removal of supraglottic structures. However, with intensive swallowing therapy, many regain functional deglutition over time. After total laryngectomy, the risk of aspiration is generally eliminated due to the complete separation of the airway and digestive tract, but patients may still face challenges related to bolus propulsion and coordination [83].

Respiratory function can be compromised by both surgical and non-surgical treatments. In total laryngectomy, the establishment of a permanent tracheostoma alters normal airway dynamics and filtration, exposing patients to environmental irritants and increasing susceptibility to respiratory infections. Pulmonary hygiene becomes more dependent on patient adherence to stoma care and humidification. In contrast, patients undergoing CRT may develop subglottic stenosis, edema, or impaired laryngeal mobility, which may require temporary tracheostomy or airway interventions [84].

Nutritional status is tightly linked to functional outcomes. Dysphagia, xerostomia, taste changes, and odynophagia contribute to reduced oral intake and weight loss, particularly in patients undergoing chemoradiation. These deficits can lead to sarcopenia, fatigue, and poor wound healing. In contrast, post-laryngectomy patients often resume oral intake more quickly, albeit with limitations in taste and olfactory feedback due to disrupted airflow through the nasal cavity. Communication efficiency, a function distinct from voice production alone, is significantly impaired after total laryngectomy. Patients must learn to use alternative speech methods, such as tracheoesophageal speech, esophageal speech, or electrolarynx devices. These methods require variable degrees of motor coordination, cognitive engagement, and support, influencing the speed and extent of communicative reintegration. Finally, fatigue and energy levels are commonly affected by all treatment modalities, though mechanisms differ. In chemoradiotherapy, fatigue is driven by systemic inflammation and cumulative toxicity, while in surgical patients, it is more often related to altered respiratory mechanics or nutritional deficits. Persistent fatigue can limit participation in rehabilitation and contribute to social withdrawal [85].

The functional consequences of laryngeal cancer treatment differ substantially depending on the chosen modality. Table 3 summarizes the comparative impact of total laryngectomy, radiotherapy, and chemoradiotherapy across key domains such as voice preservation, swallowing function, respiratory adaptation, nutritional risk, and rehabilitation complexity, as reported in recent clinical and longitudinal studies [9,11,28,63,64,65].

### 4.3. Rehabilitation Opportunities and Limitations by Modality

Rehabilitation in laryngeal cancer care is a critical yet often underappreciated component of recovery, directly influencing functional reintegration and long-term independence. While all treatment modalities require supportive strategies, the scope, complexity, and success of rehabilitation efforts vary significantly depending on the nature of the intervention. Tailoring rehabilitation to the patient’s treatment profile is essential for maximizing outcomes, reducing complications, and restoring autonomy [11].

In patients treated with total laryngectomy (TL), rehabilitation is highly structured and begins with voice restoration. Tracheoesophageal speech via a voice prosthesis is generally regarded as the most effective method, offering superior intelligibility and fluency compared to esophageal speech or electrolarynx use. However, the success of tracheoesophageal prostheses depends on access to surgical expertise, device availability, and long-term follow-up for maintenance and troubleshooting. In resource-limited settings, prosthetic voice rehabilitation may be unavailable or unaffordable, leading to suboptimal outcomes and communication barriers. Moreover, beyond speech, TL patients require stoma care education, pulmonary hygiene adaptation, and often psychological support to address the transition to a new respiratory route. Rehabilitation after TL also includes swallowing training, particularly for patients who experience coordination difficulties or reduced esophageal propulsion. While aspiration risk is typically eliminated by the anatomical separation of the airway and digestive tract, functional dysphagia can still occur and may be compounded by postoperative fibrosis or adjuvant radiotherapy [86].

Unlike total laryngectomy patients, those treated with radiotherapy or chemoradiotherapy retain the anatomical structures of the larynx but often experience significant functional impairments. Rehabilitation focuses on managing prolonged inflammation, fibrosis, and neuromuscular dysfunction that affect voice, swallowing, and breathing. Voice therapy aims to optimize residual vocal quality, while swallowing rehabilitation is typically longer and more complex, involving compensatory strategies, dietary adjustments, and sometimes gastrostomy feeding. Due to compromised airway protection, the risk of aspiration remains high [87].

In patients undergoing chemoradiotherapy, therapy fatigue is a major barrier, as persistent exhaustion and mucosal sensitivity can delay or limit rehabilitation efforts, potentially compromising recovery. Moreover, access to therapists experienced in post-CRT dysfunction is uneven. Across all treatment types, systemic and geographic disparities impact rehabilitation—urban centers often provide multidisciplinary care, while rural patients may face limited access, delays, or insufficient specialist availability. Insurance coverage restrictions further reduce the continuity and effectiveness of these services [88].

In summary, the potential and limitations of rehabilitation in laryngeal cancer are closely linked to the treatment pathway. While total laryngectomy allows for structured voice prosthesis programs and a clearer rehabilitation algorithm, organ-preserving modalities introduce functional unpredictability that requires flexible, individualized therapy. Addressing these differences proactively through multidisciplinary planning and equitable resource allocation is essential for optimizing recovery.

### 4.4. Patient Preferences and Shared Decision-Making

As treatment strategies for laryngeal cancer become more diverse and nuanced, the role of the patient in the therapeutic decision-making process has gained increasing prominence. Shared decision-making (SDM) is a collaborative model that integrates medical expertise with the patient’s individual values, lifestyle, and personal goals. Particularly in laryngeal cancer—where multiple modalities may offer comparable oncologic outcomes but differ substantially in functional and psychosocial consequences—patient preferences play a pivotal role in achieving personalized and satisfactory care. Unlike treatment scenarios in which one modality is clearly superior, many early- to intermediate-stage laryngeal cancers can be approached with either surgery or radiotherapy. While the clinician may outline the benefits and risks of each option, the optimal decision often depends on what the patient values most: voice preservation, treatment duration, the avoidance of visible anatomical change, or long-term function. For instance, a patient whose profession depends on vocal clarity may favor a non-surgical approach, even at the cost of longer recovery or potential late toxicity, whereas another may prioritize definitive tumor removal and accept the consequences of total laryngectomy [73].

The effectiveness of shared decision-making (SDM) depends on clear, balanced communication about the treatment outcomes, functional impacts, and quality of life considerations. Tools like brochures, patient stories, and digital aids can help patients better understand their options. Emotional readiness also plays a key role; early support from psycho-oncology professionals or patient navigators can aid overwhelmed patients in processing information and making informed choices. Cultural values, health literacy, and family involvement influence treatment preferences and must be respected to ensure trust and alignment with the patient’s coping style. Additionally, personal experiences with illness may shape priorities, highlighting the need for individualized, empathetic discussions [89].

Decisional regret is a documented phenomenon in laryngeal cancer patients and is more likely to occur when expectations are misaligned with outcomes or when patients feel excluded from the decision-making process. Preventing this requires not only technical expertise but also listening skills, empathy, and longitudinal support.

Ultimately, shared decision-making fosters a therapeutic alliance rooted in mutual respect and realistic hope. It transforms the patient from a passive recipient of care into an informed participant whose values actively shape the treatment trajectory. In laryngeal cancer—where physical changes are profound and personal identity is often challenged—this approach is not only ethical but essential for achieving meaningful recovery [90].

In conclusion, the type of treatment administered in laryngeal cancer exerts a profound influence not only on survival but also on functional outcomes, psychological adaptation, and long-term reintegration. Each modality—surgical or non-surgical—presents a distinct profile of benefits, risks, and rehabilitative demands, with significant variability in patient experience. Effective management requires more than a technical understanding of oncologic protocols; it demands personalized guidance, anticipatory planning, and shared decision-making that honors the patient’s voice—both literally and metaphorically. As treatment-related functional and emotional consequences unfold over time, equitable access to tailored rehabilitation becomes essential to recovery. However, despite these known needs, significant gaps remain in the psychological support systems available to this patient population. The next section will explore the barriers to psychological care in laryngeal cancer, addressing structural, institutional, and interpersonal factors that limit access to comprehensive support.

## 5. Barriers to Psychological Care and Support

Although the psychological burden of laryngeal cancer is well recognized, the real-world implementation of adequate psychological care remains limited. Several barriers—systemic, cultural, and individual—prevent patients from receiving timely and appropriate support.

At the institutional level, psychological care is frequently undervalued in oncologic protocols, which remain primarily focused on tumor control and physical rehabilitation. Mental health services are often not embedded into head and neck cancer care pathways and may be considered ancillary rather than essential. In many hospitals, psycho-oncology units are either understaffed or entirely absent, leaving oncologists and surgeons with limited resources to address the emotional needs of their patients. Time constraints during consultations and a lack of interdisciplinary coordination further reduce the likelihood that psychological concerns will be identified and addressed in a timely manner. Geographical and resource disparities also play a significant role. Patients living in rural or underserved areas often have limited access to specialized mental health professionals trained in oncology-related distress. Even when psychological support is available, it may require travel, out-of-pocket costs, or long wait times—all of which discourage utilization. These structural inequalities are compounded in lower-income countries or in healthcare systems where psychosocial care is not covered by insurance or public health policies [90].

Stigma and cultural beliefs remain powerful deterrents to seeking mental healthcare, particularly among older adults or those from conservative communities. In many cultures, emotional suffering is either minimized or internalized, and expressing vulnerability may be perceived as weakness. In the context of laryngeal cancer—where patients may already feel physically diminished or socially marginalized—admitting to psychological difficulties can exacerbate feelings of shame or inadequacy. As a result, patients may underreport symptoms or decline referrals even when distress is evident. At the individual level, several psychological and practical factors interfere with help-seeking behavior. Patients overwhelmed by the physical demands of treatment or experiencing fatigue, pain, or communication barriers may not have the energy or means to engage with mental health services. Those with reduced health literacy may struggle to recognize psychological symptoms or understand the benefits of therapy. Moreover, emotional numbing, denial, or avoidance—common coping mechanisms in cancer patients—can delay or prevent the acknowledgment of psychological needs [87].

Another important but often overlooked barrier is the lack of systematic screening. Many institutions do not routinely administer validated tools such as the Distress Thermometer, Hospital Anxiety and Depression Scale (HADS), or other brief measures, resulting in missed opportunities for early detection. When screening does occur, it is often administered without a clear referral pathway or continuity of care, limiting its clinical impact. Psychological symptoms may also be misattributed to treatment side effects or dismissed as transient reactions, leading to underdiagnosis and undertreatment. Communication challenges inherent to laryngeal cancer can further isolate patients and complicate mental health assessment. Those who have undergone total laryngectomy may struggle to express complex emotional states or to engage in traditional talk therapy. The physical limitations imposed by voice loss or altered speech mechanics necessitate adaptation by therapists and institutions, yet such flexibility is rarely available in standard care models [91].

Multiple barriers continue to prevent the effective integration of psychological services in the care of laryngeal cancer patients. These obstacles span from institutional and systemic limitations to sociocultural and individual-level challenges. Table 4 categorizes these barriers and illustrates examples drawn from the current literature [42,43,47,52,53].

Beyond logistical support, caregivers of laryngeal cancer patients frequently experience significant psychological distress, often mirroring the emotional burden of the patient. Studies show high levels of anxiety, fatigue, and depressive symptoms among caregivers, particularly when communication becomes impaired or when rehabilitation demands are high. Including caregivers in support programs and offering dedicated counseling can improve both caregiver well-being and patient outcomes [7,92].

In summary, barriers to psychological care in laryngeal cancer are multifactorial and deeply embedded in both the healthcare system and sociocultural context. Overcoming these obstacles requires a paradigm shift that positions mental health as an integral component of cancer care, supported by infrastructure, training, policy, and empathy. Proactive screening, culturally sensitive communication, multidisciplinary collaboration, and equitable access to services are essential steps toward ensuring that no patient endures the psychological consequences of laryngeal cancer in silence.

## 6. Recommendations for Integrated Survivorship Care

The transition from active treatment to survivorship in laryngeal cancer is often marked by persistent functional and psychological challenges. To improve long-term outcomes, survivorship care should adopt a multidisciplinary, patient-centered approach that extends beyond oncologic surveillance.

A key priority is routine screening for psychological distress using validated tools such as the Hospital Anxiety and Depression Scale (HADS) or Distress Thermometer at defined intervals. Early detection allows for timely referral to psycho-oncology services and supports tailored interventions. Multidisciplinary survivorship clinics are recommended, integrating oncologists, speech therapists, psychologists, and dietitians to coordinate post-treatment care, including voice and swallowing rehabilitation [93]. Remote solutions such as tele-rehabilitation may help bridge accessibility gaps, especially in underserved areas. Attention should also be given to informal caregivers, who often face emotional strain. Providing them with information and psychological support can strengthen the patient’s recovery environment. Lastly, patient education and shared decision-making should remain central to the survivorship model, empowering individuals to participate in rehabilitation planning and to manage long-term risks. Institutional support, reimbursement policies, and further research into survivorship outcomes are essential to sustain these efforts [22,94].

To enhance the feasibility of these recommendations, specific implementation strategies should be adopted at the institutional and system levels. First, training programs for clinicians, including ENT surgeons, oncologists, and nurses, should be developed to increase awareness of psychological morbidity, facilitate the use of validated screening tools (e.g., HADS and Distress Thermometer), and support referral to mental health services. Interdisciplinary workshops and continuous medical education (CME) modules can foster collaboration between oncologic and psycho-oncologic teams [95].

Second, institutions should create structured care pathways integrating psychological assessment at predefined time points (e.g., diagnosis, post-treatment, and follow-up visits). Embedding these tools into electronic medical records can streamline screening and flag high-risk patients.

Third, healthcare systems should support the development of dedicated survivorship coordinators or patient navigators trained to monitor psychosocial needs and facilitate access to rehabilitation, speech therapy, and counseling services. These roles have shown effectiveness in other oncologic contexts and could bridge existing gaps in head and neck cancer survivorship care.

In parallel, technological solutions can play a critical role in mitigating barriers to psychological care, especially in low-resource or rural areas. Mobile health (mHealth) applications designed for mental health screening, mood tracking, and guided cognitive-behavioral therapy (CBT) have shown promise in oncology populations. These platforms can provide asynchronous support, reduce stigma, and promote self-monitoring of distress. Examples include apps incorporating psychoeducation, relaxation techniques, and alert systems for clinical referral [96].

Additionally, telepsychiatry and tele-rehabilitation programs have proven feasible in head and neck cancer patients, enabling real-time psychological counseling, voice therapy, and caregiver education. Integrating these tools into survivorship care plans could improve follow-up adherence and the continuity of support in geographically underserved settings. Further research is needed to validate and culturally adapt such platforms for patients with laryngeal cancer, particularly those with post-treatment communication limitations [97].

To operationalize a patient-centered survivorship model for individuals treated for laryngeal cancer, several domains must be addressed in a coordinated and anticipatory manner. Table 5 outlines the key components of integrated follow-up care, including routine psychological screening, functional rehabilitation, education, and caregiver support. These recommendations are grounded in existing evidence highlighting the importance of continuous assessment, multidisciplinary involvement, and patient empowerment [11,28,42,47,52].

In conclusion, integrated survivorship care in laryngeal cancer should be anticipatory, interdisciplinary, and adaptive, addressing not only medical surveillance but also the full range of functional and psychosocial sequelae. By adopting a holistic and proactive approach, healthcare systems can better support survivors in reclaiming their identity, autonomy, and quality of life after cancer.

## 7. Limitations

This review has several limitations inherent to its narrative design. Unlike systematic reviews, it does not follow a predefined search protocol or include a formal risk-of-bias assessment. Consequently, the selection of sources may be subject to publication bias and selection bias, with a higher likelihood of including studies that report significant psychological or HRQoL outcomes.

Additionally, the heterogeneity of study designs, instruments used (e.g., HADS, EORTC QLQ-H&N35, and VHI), and population characteristics limit direct comparability across cited data. The review also lacks meta-analytic synthesis, which restricts the ability to quantify effect sizes or assess statistical heterogeneity. Although this review does not strictly follow the PRISMA guidelines, its development was informed by the PRISMA 2020 for Narrative Reviews framework, with an emphasis on transparency, structured presentation, and comprehensive coverage of relevant studies in the literature.

While every effort was made to include recent and clinically relevant studies in the literature, the absence of a systematic search strategy may have resulted in the omission of pertinent studies. Finally, most data are drawn from high-income countries, limiting generalizability to low-resource settings or culturally diverse populations. These limitations should be considered when interpreting the findings and recommendations presented herein.

## 8. Conclusions

Laryngeal cancer imposes a complex and enduring burden that extends well beyond tumor eradication. Its treatment—whether surgical or non-surgical—alters vital functions such as voice, swallowing, and breathing, impacting not only physical capabilities but also psychological well-being, social engagement, and personal identity. This review has highlighted the high prevalence of anxiety and depression among laryngeal cancer patients, the variable and often underappreciated effects of treatment modalities on quality of life, and the multifactorial influences that shape survivorship experiences.

Despite the increasing recognition of these challenges, significant gaps remain in the delivery of holistic, patient-centered care. Barriers to psychological support, inconsistent rehabilitation access, and fragmented survivorship planning limit the potential for full functional and emotional recovery. Integrating standardized psychological screening, multidisciplinary rehabilitation, and shared decision-making into routine oncologic care is not merely an enhancement—it is a necessity for restoring dignity, autonomy, and well-being in this vulnerable population.

A shift in paradigm is urgently needed—one that views survival not as the end point but as the beginning of a broader recovery journey. Tailored, equitable, and proactive support strategies must become standard practice in laryngeal cancer care, ensuring that every patient receives not only life-extending treatment but also the tools to reclaim a meaningful and connected life after cancer.

## Figures and Tables

**Table 1:** Summary of selected studies reporting anxiety and depression prevalence in laryngeal/head and neck cancer patients.

Study (Author, Year)	Study Design	Sample Size	Population	Tool Used	Main Findings
Jiménez-Labaig et al., 2025 [14]	Meta-analysis	–	HNC patients (multiple subsites)	–	Anxiety: 29.9% baseline; 17.4% later stages
Rohde et al., 2018 [18]	National registry study	71,541	HNC; laryngeal subgroup: 28.5% MDD	ICD-10 diagnosis codes	Laryngeal cancer = highest prevalence
Perry et al., 2015 [9]	Longitudinal	32	TL patients	HADS, EORTC QLQ-H&N35	Improved psychosocial scores with voice rehab
Johansson et al., 2011 [10]	Longitudinal	100	Laryngeal cancer	MAC, HADS	Mental adjustment correlated with survival
Zhang et al., 2020 [13]	Interventional	84	Laryngeal cancer	Depression scale (unnamed)	Psychological services reduce depression

**Table 2:** Quality of life instruments and reported scores in selected studies.

Study (Author, Year)	Instrument Used	Population	Key Domains Assessed	Main QoL Findings
Johansson et al., 2011 [10]	EORTC QLQ-H&N35, HADS	TL patients	Speech, senses, emotional function	Lower speech and emotional scores; persistent anxiety
Perry et al., 2015 [9]	EORTC QLQ-H&N35, HADS	TL patients	Social contact, self-efficacy	Gradual improvement with voice rehab
Bergström et al., 2017 [11]	VHI, HADS	Post-TL patients	Voice handicap, depression	Improved VHI scores with structured voice therapy
Trivic et al., 2016 [5]	EORTC QLQ-H&N35	Serbian TL patients	Speech, swallowing, pain	Validated tool; high internal consistency
Zhang et al., 2020 [13]	Depression scale (unnamed)	Laryngeal cancer	Depression	Psychological support reduced symptom scores

**Table 3:** Comparative overview of functional outcomes and rehabilitation needs in laryngeal cancer patients according to treatment modality. Data are synthesized from published clinical studies and reviews.

Treatment Modality	Voice Function	Swallowing Function	Respiratory Consequences	Nutritional Risk	Rehabilitation Complexity
Total Laryngectomy	Absent—requires prosthetic	Generally preserved	Requires tracheostoma	Moderate	Structured, device-based
Radiotherapy	Preserved but may be hoarse	May develop fibrosis/dysphagia	Minimal direct effect	Moderate to high	Variable
Chemoradiotherapy	Variable—vocal fatigue common	High risk of chronic dysphagia	Potential airway edema	High	Prolonged, complex

**Table 4:** Classification of barriers to psychological care in laryngeal cancer patients with representative examples. Adapted from published studies addressing structural, interpersonal, and cultural limitations in psycho-oncology.

Barrier Type	Description	Examples
Institutional	Structural limitations in oncology systems	Lack of embedded psycho-oncology teams; limited consultation time [42]
Geographical/Access	Resource disparities based on location or infrastructure	Rural areas without specialized services; travel burden; long waitlists [43]
Cultural/Stigma	Social norms that discourage mental health engagement	Emotional suppression; fear of being seen as weak; stigma in older adults [53]
Individual	Psychological or practical barriers to help-seeking	Denial; fatigue; communication deficits; low health literacy [47,52]
Screening/Pathway	Lack of systematic identification and referral	No routine HADS or distress scale; unclear referral flow; underdiagnosis [42]
Caregiver-Related	Limited support for caregivers affecting patient support	Untrained or overwhelmed caregivers; no caregiver-centered programs [52]

**Table 5:** Core components of integrated survivorship care in laryngeal cancer. Recommendations are based on published evidence emphasizing routine psychological evaluation, rehabilitation continuity, caregiver inclusion, and patient engagement.

Domain	Recommended Intervention	Implementation Strategy
Psychological screening	Routine HADS or distress scale	Baseline + every 3–6 months
Voice rehabilitation	Tracheoesophageal speech, voice therapy	Early initiation + device access
Swallowing therapy	Videofluoroscopic assessment + exercises	Long-term monitoring
Patient education	Tailored materials, digital modules	During treatment and follow-up visits
Caregiver support	Training, counseling options	Integrated into family appointments
